# Noise reduction of electron holography observations for a thin-foiled Nd-Fe-B specimen using the wavelet hidden Markov model

**DOI:** 10.1186/s42649-024-00097-w

**Published:** 2024-04-17

**Authors:** Sujin Lee, Yoshihiro Midoh, Yuto Tomita, Takehiro Tamaoka, Mitsunari Auchi, Taisuke Sasaki, Yasukazu Murakami

**Affiliations:** 1 Department of Applied Quantum Physics and Nuclear Engineering, Kyushu University, Fukuoka, 819-0395 Japan; 2 Present address: Korea Institute of Materials Science, Changwon, 51508 Korea; 3 Graduate School of Information Science and Technology, Osaka University, Osaka, 565-0871 Japan; 4 The Ultramicroscopy Research center, Kyushu University, Fukuoka, 819-0395 Japan; 5 National Institute for Materials Science, Tsukuba, 305-0047 Japan

**Keywords:** Noise reduction, Wavelet hidden Markov model, Electron holography, Nd-Fe-B magnet

## Abstract

In this study, we investigate the effectiveness of noise reduction in electron holography, based on the wavelet hidden Markov model (WHMM), which allows the reasonable separation of weak signals from noise. Electron holography observations from a Nd_2_Fe_14_B thin foil showed that the noise reduction method suppressed artificial phase discontinuities generated by phase retrieval. From the peak signal-to-noise ratio, it was seen that the impact of denoising was significant for observations with a narrow spacing of interference fringes, which is a key parameter for the spatial resolution of electron holography. These results provide essential information for improving the precision of electron holography studies.

## Introduction

Off-axis electron holography, a method related to transmission electron microscopy (TEM), determines the phase shift of an electron wave traversing a thin-foil specimen (Völkl et al. 1999; Tonomura 1999; Midgley 2001). As described in detail later, the phase of the object wave can be retrieved from the electron hologram produced by interference with the reference wave. Since the phase shift in the object wave originates from the electromagnetic field of the specimen, the reconstructed phase image approximates the map of the electrostatic potential and/or magnetic induction (*i.e.*, the in-plane component of the magnetic flux density, ***B***). Because of this functionality, electron holography has been applied to the mapping of electromagnetic fields in p-n junctions of semiconductor devices (Rau et al. 1999; Wang et al. 2002), permanent magnets (McCartney and Zhu 1998; Zhu et al. 2001; Murakami et al. 2014), magnetic nanoparticles (Tonomura et al. 1980; Tripp et al. 2003; Yamamoto et al. 2007; Ammar et al. 2008; Takeno et al. 2014; Biziere et al. 2019), magnetic skyrmions (Park et al. 2014; Kovács et al. 2017; Shibata et al. 2017), magnetic fluxons in superconductors (Matsuda et al. 1991; Harada et al. 1996), and several other materials. As shown by Lichte and Lehman (Lichte and Lehmann 2008), the precision of the phase analysis of electron holography depends on the image quality of the electron holograms, which comprise interference fringes of the object and reference electron waves. Indeed, the phase-detection limit can be explained by factors such as the number of electrons per resolved pixel, detection quantum efficiency of the camera, and fringe contrast of the electron hologram (*i.e.*, the visibility, ${V}_{obs}$) (Harscher and Lichte 1996; Ruijter and Weiss 1993).

A noise reduction method based on image processing that can be applied to either an electron hologram or reconstructed phase image is effective for improving the accuracy of the phase analysis. As a noise-reduction strategy applicable electron holograms, Anada et al. (Anada et al. 2019; Anada et al. 2020) demonstrated the usefulness of sparse coding, which clearly visualized the electrostatic potentials of p-n junctions in GaAs-based semiconductor devices. Nomura et al. (Nomura et al. 2021) employed tensor decomposition to derive the phase information from electron holograms acquired under low-electron-dose conditions. Another effective noise-reduction method is the wavelet hidden Markov model (WHMM) developed by Midoh and Nakamae (Midoh and Nakamae 2020; Miura Lab 2023). The WHMM performs noise reduction using a wavelet transform by depressing the wavelet coefficients statistically. In the conventional thresholding method of the wavelet coefficients, the application of a threshold eliminates not only noise, but also weak signals lower than the threshold limit (Jansen 2012). To solve this problem, Midoh and Nakamae expressed a process of wavelet transform using Markov parameters (Midoh and Nakamae 2020). To briefly explain WHMM, we suppose two hidden states *L* and *S*, which are related to the production of signal and noise, respectively. Regarding the two hidden states, we define the parameters ${\sigma }_{L}$ and ${\sigma }_{S}$ for each image pixel in all frequency levels of the wavelet transform. With reference to the hidden state *L*, the parameter ${\sigma }_{L}$ represents that an image pixel produces the wavelet coefficient *w* (determined for all the image pixels by using observations) with probability ${\sigma }_{L}$. Similarly, the parameter ${\sigma }_{S}$ represents the probability to produce *w* with reference to the hidden states *S*. The transition probability $\varepsilon$ is used to explain the feature of hidden states (as to which state is dominant in one image pixel) can be inherited to the other pixel at the lower/upper frequency level during the forward/inverse wavelet transform. The $\varepsilon$ should be defined for all the image-pixel pairs (for corresponding pixels between upper and lower frequency levels). These parameters are optimized by the Baum-Welch algorithm, so that the observations of *w* can be most reasonably explained by the model. In other words, the WHMM offers optimized noise reduction for individual image pixels depending on the probability of representing the signal or noise. We refer the reader to the original paper (Midoh and Nakamae 2020) for more details on WHMM.

Midoh and Nakamae (Midoh and Nakamae 2020) discussed the usefulness of the WHMM using artificially calculated electron holograms. Regarding holograms obtained experimentally, Tamaoka et al. (Tamaoka et al. 2021) applied WHMM to observations from a non-magnetic, multiple-layered film of LaFeO_3_/SrTiO_3_. Noise reduction improved the quality of the reconstructed phase image, representing a gap in the electrostatic potential at the LaFeO_3_/SrTiO_3_ interface. From these results, it is expected that WHMM can be widely applied to electron holography observations. One of the essential targets is magnetic domain observation from a permanent magnet, including an Nd_2_Fe_14_B crystal. Due to the large magnetocrystalline anisotropy in Nd_2_Fe_14_B crystals, Nd-Fe-B-based magnets are the strongest commercial magnet widely used for traction motors in electric vehicles and several other applications (Hono and Sepehri-Amin 2012). Magnetic domain analysis is vital for improving the performance of Nd-Fe-B-based magnets. However, in studies using TEM, the Nd_2_Fe_14_B crystal provides poor image contrast due to the significant electron absorption by the heavy element Nd. Indeed, it appears that the visibility of electron holograms from Nd_2_Fe_14_B crystals is poor compared to observations from other magnetic alloys and compounds. For example, an Fe_70_Al_30_ alloy allowed magnetic flux mapping over a wide range of the foil thickness up to 230 nm with an acceleration voltage of 300 kV (Murakami et al. 2014). However, in the previous electron holography study of Nd_2_Fe_14_B magnet, the magnetic flux density was determined using a thin-foiled specimen the thickness of which was approximately 100 nm in the acceleration voltage of 300 kV (Murakami et al. 2014). Thus, noise reduction is essential for precision improvement in magnetic domain analysis.

**Fig. 1:**
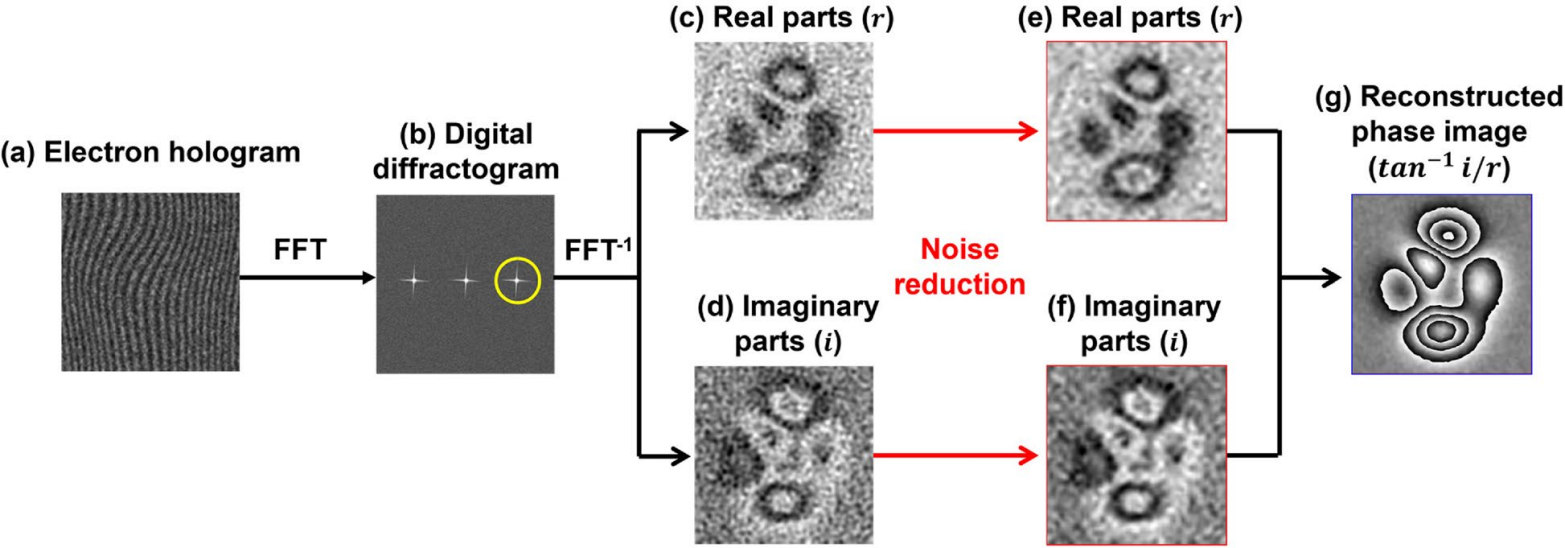
Process of phase retrieval. **a** Electron hologram. **b** Digital diffractogram obtained by fast Fourier transform (FFT). The yellow circle in (**b**) indicates a selected specific frequency zone for FFT^-1^ of the diffractogram. **c** and **d** Real and imaginary parts of a complex image reconstructed from the hologram *via* FFT^-1^. **e** and **f** Results of real and imaginary parts after applying the noise reduction to (**c**) and (**d**), respectively. **g** Reconstructed phase image obtained by calculating $\tan^{-1}i/r$, where $i$ and $r$ are the imaginary part of (**f**) and the real part of (**e**), respectively

The spacing of the interference fringes (*s*) in the hologram is generally of the order of 1 nm, as shown in the subsequent section. Narrow fringe spacing is essential for high spatial resolution in electron holography (Völkl et al. 1999). However, as a feature of noise reduction using a wavelet transform (including the application of the WHMM), the high-frequency component related to the narrow fringe spacing is more depressed than the low-frequency component related to wide-spacing fringes. To address this issue, an alternative method of noise reduction involves applying the WHMM to the real (*r*) and imaginary (*i*) parts of the complex image produced via a fast Fourier transform (FFT) using the electron hologram (Fig. 1c and d). Indeed, in the previous studies using WHMM (Midoh and Nakamae 2020; Tamaoka et al. 2021), the noise reduction was applied to the electron holograms which were made of interference fringes of electron waves. In contrast, in this study, the noise reduction is applied to the complex images. The phase-retrieval process shown in Fig. 1 is discussed in further detail later.

Thus, the purpose of this study is to evaluate the usefulness of noise reduction by WHMM, for which the noise reduction was applied to the real and imaginary parts of the complex image in the real space. Electron holograms were acquired from a thin-foil specimen comprising Nd_2_Fe_14_B crystals.

## Experimental method

**Fig. 2:**
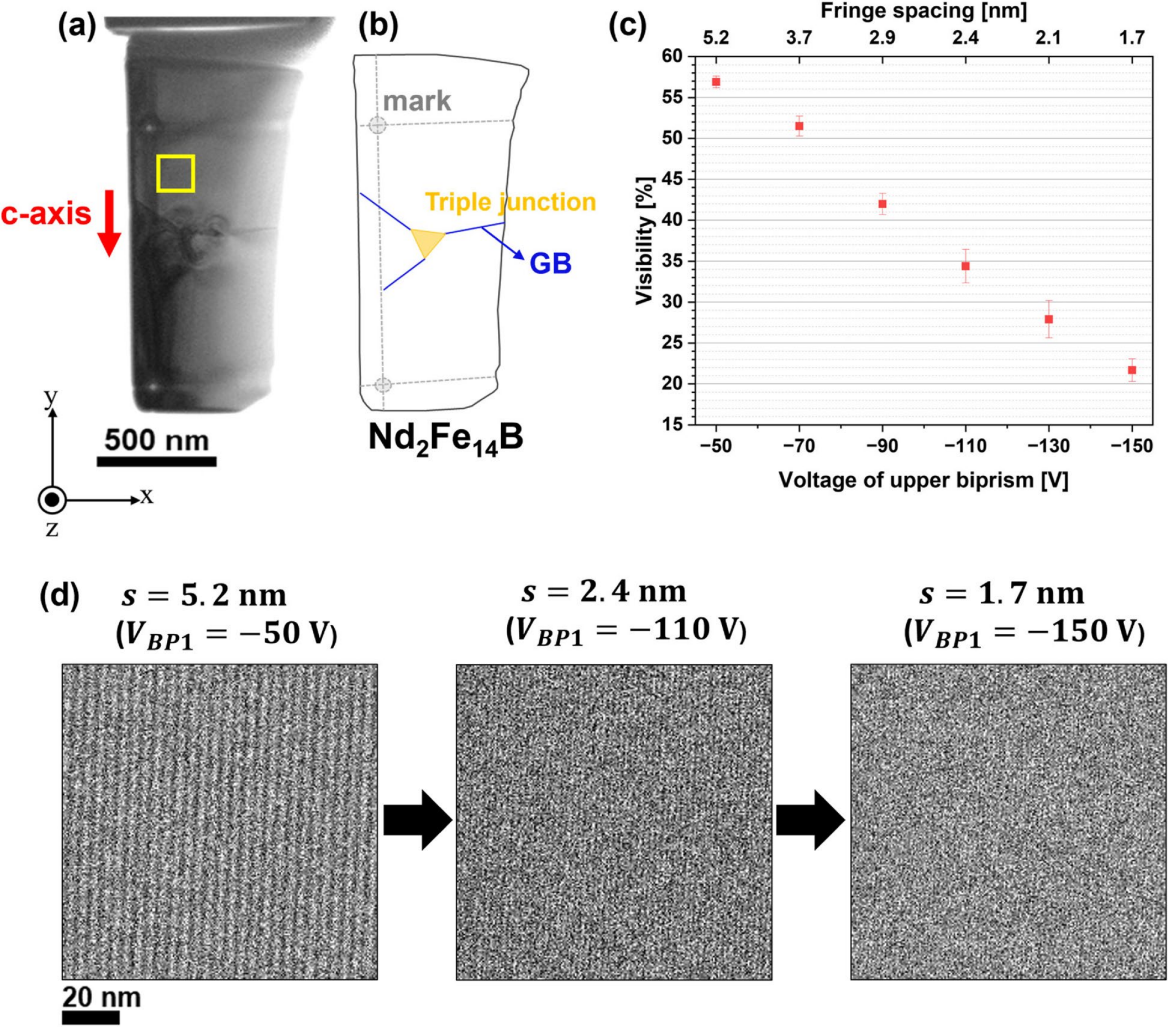
Observation of a thin-foiled Nd_2_Fe_14_B specimen by electron holography. **a** TEM image of the Nd_2_Fe_14_B specimen. The yellow rectangular area in (**a**) indicates the field view for electron holograms in (**d**). **b** Schematic of the thin-foiled specimen. **c** Visibility of electron holograms as a function of the voltage of upper biprism (lower horizontal axis) and fringe spacing (upper horizontal axis). **d** Series of electron holograms acquired at fringe spacings of 5.2 nm, 2.4 nm, and 1.7 nm, corresponding to *V*_*BP1*_ of $-$50 V, $-$ 110V, and $-$150 V, respectively

A thin-foil Nd_2_Fe_14_B specimen was used to evaluate the denoising effect using a WHMM. A focused ion beam/scanning electron microscope (FIB/SEM; Helios G4 UX, FEI Co.) was used to obtain a polycrystalline rectangular Nd-Fe-B block, which was polished into a thin foil using another FIB instrument (MI4000L, Hitachi Ltd.). To reduce surface damage, the foil was polished at low acceleration voltages (5 kV and 2 kV) using a Ga-ion beam during the final stage of specimen preparation. Figure 2a shows the TEM image of the Nd_2_Fe_14_B thin-foil specimen. The specimen contained grain boundaries and a triple junction comprising an Nd-rich phase, as shown schematically in Fig. 2b. The grey circles in Fig. 2b indicate the positions of the artificially produced dimples that were referred to in the thinning process; the grey dotted lines offer a guide to visualize the positions of the dimples. Since the specimen was an isotropic magnet, the directions of the *c*-axis in the three Nd_2_Fe_14_B grains were approximately parallel to those indicated by the red arrow. With reference to the *x-y-z* coordinate system in Fig. 2a, the left edge of the thin-foil specimen was parallel to the *y*-axis, while the electron was incident along the $-$*z* direction. Electron holograms were acquired with various values of fringe spacing, *s*, using a 300-kV TEM (HF-3300X, Hitachi Hightech) with double-biprism electron interferometry (Harada et al. 2004). The double-biprism system enables changing the fringe spacing *s* and interference width *W* (*i.e.*, fringe spacing multiplied by the number of fringes) independently. In this study, the applied voltage of the upper biprism *V*_*BP1*_ was varied ($-$50 V, $-$70 V, $-$90V, $-$110 V, $-$130 V, and $-$150 V) to obtain several values of *s*, while the voltage of the lower biprism *V*_*BP2*_ was fixed at -100 V. As described later in detail, these conditions attained the values of *s* at 5.2 nm, 3.7 nm, 2.9 nm, 2.4 nm, 2.1 nm, and 1.7 nm, although *W* remained unchanged at 1344 nm. For all holograms, the electron exposure time (*t*_*a*_) was 3.0 s. The electron-dose rate was approximately 1.5 $\text{e}^{-}/ (\mathring{\text{A}} \cdot \text{s})$ (*i.e.*, beam current density of 24 ${\text{A}}/{{\text{m}}}^{2}$). The variation in *s* affects the visibility of the electron holograms, as shown in Fig. 2c, which is discussed in greater detail in the next section. Holograms were recorded using a high-sensitivity camera (K3 IS camera, Gatan Inc.) (Chang et al. 2016).

As the mother wavelet in the two-dimensional wavelet transform, the “Farras wavelet” was used. Figure 1 shows the process of phase retrieval using FFT and the real and imaginary parts of the reconstructed images (*i.e.*, complex images) produced by the inverse Fourier transform (FFT^-1^). An electron hologram was subjected to FFT to obtain digital diffractogram (Fig. 1a and b). A frequency-selection mask was applied to one of the sidebands that contained the phase information due to the electromagnetic field, as shown in Fig. 1b, followed by moving the selected sideband to the position of the center band. FFT^-1^ generates a complex image (reconstructed image) that can be decomposed into real ($r$) and imaginary ($i$) parts, as shown in Fig. 1c and d. In this study, denoising by the WHMM was applied to the real and imaginary parts of the images [Fig. 1e and f] instead of to the electron hologram (Fig. 1a). A phase image revealing the electromagnetic field can be reconstructed by considering the arctangent ${{\text{tan}}}^{-1}i/r$, as shown in Fig. 1g.

The phase shift ($\phi$) in the object electron wave, which can be determined by electron holography, can be expressed as follows:

1 $$\phi =\sigma \int V\left(x,y,z\right) dz-\frac{e}{\hslash }\int {A}_{z}\left(x, y, z\right) dz,$$

where $\sigma$, $e$, and $\hslash$ are the interaction constant based on the acceleration voltage of incident electrons (along *z* direction), elementary charge, and Planck’s constant divided by 2$\pi$, respectively. $V\left(x,y,z\right)$ represents the electrostatic scalar potentials. If the electric charge on the specimen is negligible, this term approximates the mean inner potential (*V*_*o*_) of the specimen, when the additional phase shift due to electron scattering in the crystal is disregarded. Indeed, to depress the undesired phase shift due to electron scattering, the specimen was tilted off a crystal zone axis to suppress the Bragg reflections. To separate the contribution of $V\left(x,y,z\right)$ [the first term in Eq. (1)] from that of ${A}_{z}\left(x, y, z\right)$[the second term in Eq. (1)], we employed the method referred to as time-reversal operation using electron waves (Tonomura et al. 1986). The time reversal can be attained by flipping the specimen upside down with respect to the incident electrons. These operation remains the electrostatic contribution to the phase shift ($\phi$) unchanged but makes the magnetic field contribution opposite sign. Finally, by subtracting the two holograms for the time reversal the phase shift only due to ${A}_{z}\left(x, y, z\right)$ can be obtained from Eq. (1). ${A}_{z}\left(x, y, z\right)$ is the *z*-component of the vector potential (***A***), which is related to the magnetic flux density (***B***) by the equation ${\varvec{B}}=rot {\varvec{A}}$. Because of this relationship, the second term in Eq. (1) determines the in-plane (*x-y* plane) component of the magnetic flux density using electron holography observations.

## Results and discussion

Figure 2c shows the visibility ${V}_{obs}$ of electron holograms as functions of the applied voltage to the upper biprism *V*_*BP1*_ (*i.e.*, lower horizontal axis) and fringe spacing *s* (*i.e.*, upper horizontal axis). As mentioned earlier, when the double-biprism system is employed, the value of *s* can be tuned by ${V}_{BP1}$, while the interference width *W* remains unchanged (Harada et al. 2004). ${V}_{obs}$ was evaluated by using Eq. (2), where ${I}_{max}$ and ${I}_{min}$ are the maximum and minimum intensities of the electron hologram (Tonomura 1999).

2 $${V}_{obs}=\frac{{I}_{max}-{I}_{min}}{{I}_{max}+{I}_{min}}$$

Figure 2c shows the average value of ${V}_{obs}$ determined for the rectangular area (framed by yellow lines) in Fig. 2a. For additional information about ${V}_{obs}$, Fig. 2d provides a series of electron holograms with *s* values of 5.2 nm, 2.4 nm, and 1.7 nm. As the fringe spacing decreases, ${V}_{obs}$ gradually decreases. Following the discussion by Chang et al. (Chang et al. 2015), the observed visibility is related to several factors, including the time-dependent part of visibility related to instrument instabilities (slower than exposure time), spatial coherence envelope of the wave field which includes instabilities faster than exposure time, and modulation transfer function (MTF) of the camera at the fringe spatial frequency ${k}_{0}$. When the electron holograms are collected at a fixed value of *V*_*BP2*_ (*i.e.*, at a constant value of *W*, which represents the spatial coherency), the decrease in visibility can be explained by the frequency dependence of the MTF (Harada et al. 2004; Chang et al. 2016). Eventually, the visibility of the electron hologram degrades when the fringe spacing is reduced, as shown in Fig. 2c and d. Note that, according to the study by Chang et al. (Chang et al. 2016), a direct detection camera (similar to the product used in this study) shows only a gradual change in MTF in a wide range of the Fourier space. The impact of the decrease in MTF, which is observed in the neighborhood of the side band, on the phase retrieval remains to be an open question.

**Fig. 3:**
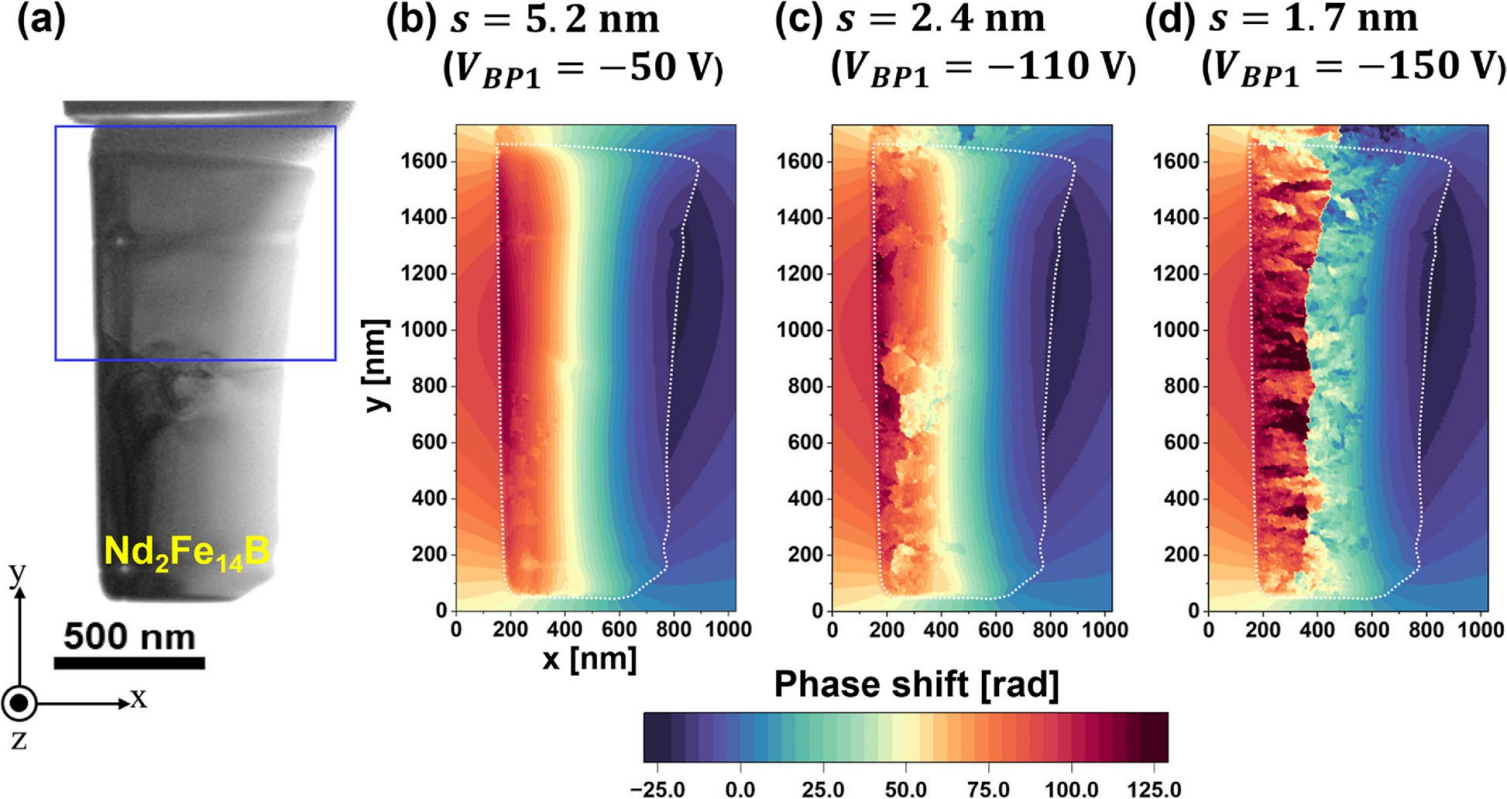
Influence of the fringe spacing on the visibility of the reconstructed phase image. **a** TEM image of the thin-foiled specimen, identical to Fig. 2a. The blue rectangular area in (**a**) indicates the area for phase images in Fig. 4. **b**-**d** Reconstructed phase images at fringe spacings of 5.2 nm, 2.4 nm, and 1.7 nm, respectively

The deterioration of ${V}_{obs}$ affects the quality of the phase image reconstructed using the FFT process, as shown in the schematic in Fig. 1. Figure 3b-d provide phase images reconstructed from the holograms acquired at fringe spacings of (b) 5.2 nm (*V*_*BP1*_ $= -$50 V), (c) 2.4 nm (*V*_*BP1*_ $= -$110 V), and (d) 1.7 nm (*V*_*BP1*_ $= -$150 V), respectively. The change in phase is represented by colors as per the color bar in Fig. 3. Phase images were obtained from the field of view shown in Fig. 3a [identical to that in Fig. 2a]. In the reconstructed phase images, the position of the specimen border is indicated by a white dotted line.

As mentioned earlier, the visibility ${V}_{obs}$ of the electron holograms was reduced by decreasing the fringe spacing, as shown in Fig. 2c and d. Importantly, when the fringe contrast of an electron hologram is poor, a phase-unwrapping algorithm (Völkl et al. 1999) that was used introduces an artificial discontinuity in phase: *i.e.*, an unwanted phase difference of 2π, which is represented by patches in the colored phase maps. The appearance of the phase jump has been briefly explained in Refs. (Völkl et al. 1999) and (Völkl et al. 1995). As explained earlier, a phase shift can be determined by calculating ${{\text{tan}}}^{-1}i/r$ using the real and imaginary parts of a complex image (Fig. 1). Due to the calculation using an arctangent function, the reconstructed phase is presented in a “wrapped” form, plotted in a limited range from -$\pi$ to $\pi$. A phase-unwrapping algorithm is employed to “unwrap” the phase image such that the phase can be plotted in an extended range [*i.e.*, a continuous change in phase can be revealed over the field of view, as shown in Fig. 3b]. For phase unwrapping, this study used a code incorporated in the commercial software HoloWorks v.5.0 (Gatan). However, the phase unwrapping was unsuccessful when the visibility ${V}_{obs}$ of the electron hologram was poor. Indeed, as shown in Fig. 3b-d, the population of patches (*i.e.*, artificial phase jumps) increased with a decrease in ${V}_{obs}$. Since the foil thickness was reduced on the right side of the specimen (showing a wedge-shaped cross-section), the fringe contrast was better than that observed on the left side. The number of patches on the right side of the specimen was negligible. However, owing to the increase in foil thickness, the fringe contrast on the left side was poor. Therefore, more patches are observed on the left side of the specimen.

**Fig. 4:**
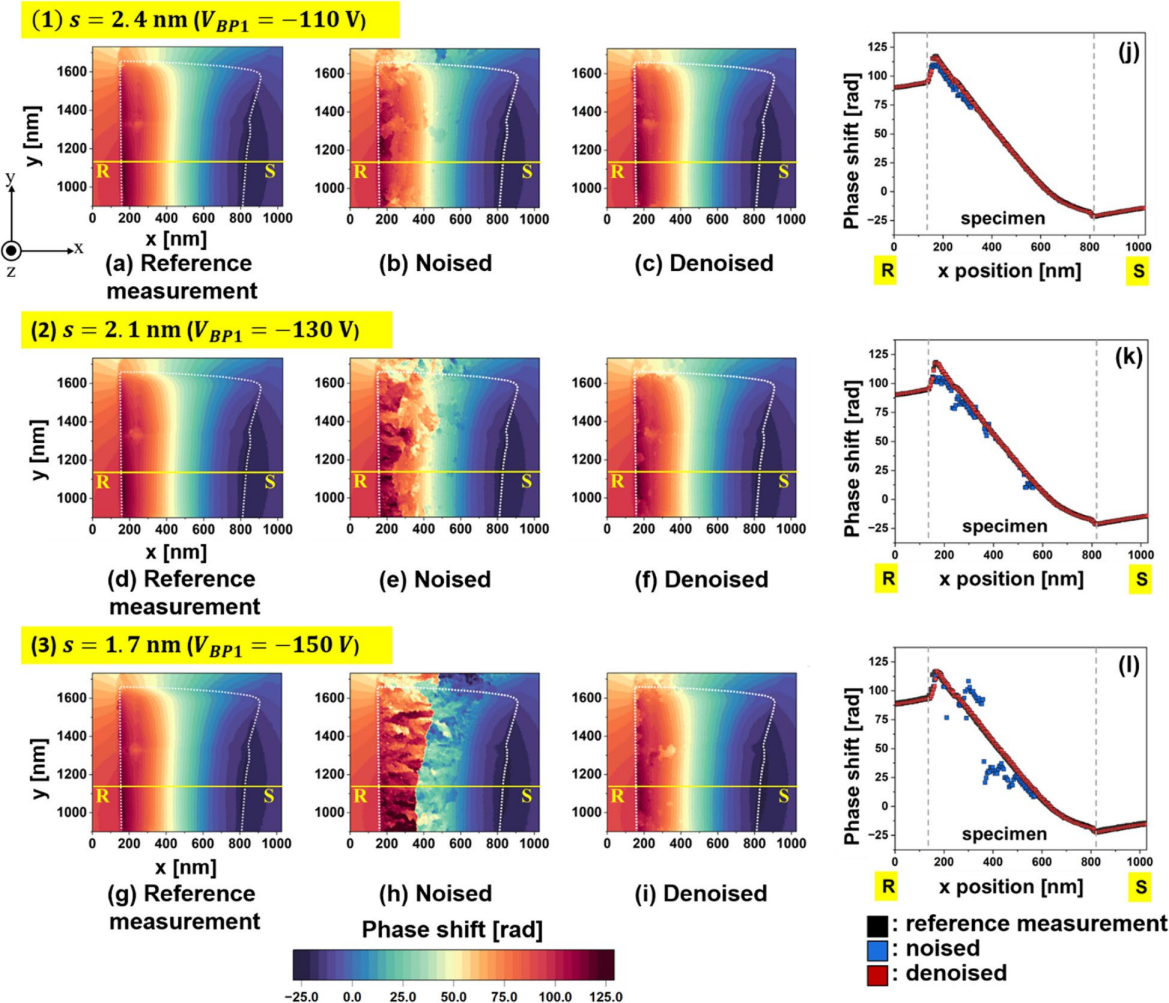
Phase image series collected with three fringe spacings: (1) *s* $=$ 2.4 nm, (2) *s* $=$ 2.1 nm, and (3) *s* $=$ 1.7 nm. **a**, **d**, and **g** Reference-measurement images with long exposure time *t*_*a*_ of 15 s for (1), (2), and (3), respectively. **b**, **e**, and **h** Original phase images, labelled “Noised,” with *t*_*a*_ of 3 s for (1), (2), and (3), respectively. **c**, **f**, and **i** Phase images, labelled “Denoised,” after the application of the noise reduction to (**b**), (**e**), and (**h**), respectively. **j**, **k**, and **l** Plots of the phase shift measured along the R–S line in (**a**)–(**c**), (**d**)–(**f**), and (**g**)–(**i**), respectively. Black, blue, and red plots in (**j**)–(**l**) indicate the results from reference-measurement image [from (**a**), (**d**), and (**g**)], noised image [from (**b**), (**e**), and (**h**)], and denoised image [from (**c**), (**f**), and (**i**)], respectively

We discuss the impact of WHMM on phase retrieval, in which denoising is applied to the real and imaginary parts of a complex image, as shown in Fig. 1. To discuss the usefulness of the WHMM, we focus on the population of artificial phase jumps (*i.e.*, patches in colored phase images), which appear to be reduced by denoising. Figure 4a–i summarize the phase images collected under three conditions of fringe spacing *s*: (a)–(c) 2.4 nm, (d)–(f) 2.1 nm, and (g)–(i) 1.7 nm. The field of view corresponds to the rectangular area shown in Fig. 3a. For each *s* series of images, the “reference measurement” represents the phase images showing only negligible patches as they were reconstructed from holograms (at the three conditions of *s*) with a sufficiently long exposure time *t*_*a*_ = 15 s; see Fig. 4a, d, and g. As revealed by the phase plots measured in the R-S line crossing the specimen (Fig. 4j, k, and l), the three reference-measurement images were almost identical in terms of the magnitude and smoothness in the phase map (black dots in (j), (k), and (l)), although they are mostly overlapped by red dots representing the noise-reduced (denoised) image, as explained later in detail. Note that the steep changes in the phase, which were observed at the specimen borders ($x=$ 170 nm and $x=$ 825 nm) were due to the contribution from the mean inner potential of the crystal. The phase shift owing to the magnetic flux density in the Nd_2_Fe_14_B crystal [*i.e.*, the second term in Eq. (1)] was superimposed on the phase shift due to the mean inner potential [*i.e.*, the first term in Eq. (1)] for electron holography observations. Nevertheless, because of the significant contribution from the magnetic flux density, the phase plot continued to decrease over the range of 170 nm $<x<$ 825 nm, in which the specimen was magnetized along the $-$ *y* direction. Reference-measurement images were used to quantify the denoising effect.

The images labeled “noised” represent the original phase images to which the noise reduction by WHMM was not applied; see Fig. 4b, e, and h. Since the exposure time was only 3 s (*i.e.*, the visibility of the interference fringe was poor), many patches were produced during the phase retrieval process, particularly in Fig. 4e and h. With respect to the original images labeled “noised,” the phase shift was measured along the R-S line, where the position was identical to that of the reference-measurement images. The results are indicated by the blue dots in Fig. 4j for *s* $=$ 2.4 nm, Fig. 4k for *s* $=$ 2.1 nm, and Fig. 4l for *s* $=$ 1.7 nm. In the presence of several patches in the original images, the blue dots deviated from the reference-measurement curve. The deviation was especially large in the observation at *s* $=$ 1.7 nm since the visibility of electron hologram was the lowest. Additionally, as mentioned earlier, the deviation from the reference-measurement curve was significant on the left side of the specimen compared to that on the right side, because the visibility was reduced in the thick portion of the specimen.

Figure 4c, f, and i, labeled “denoised,” show the phase images to which denoising by WHMM was applied. As a result of the noise reduction applied to the real and imaginary parts of the complex image (Fig. 1), the patches in the phase images were mostly eliminated in all observations. Regarding the noise-reduced (denoised) images, the plots of the phase shift measured along the R-S line are shown in red in Fig. 4j for *s* $=$ 2.4 nm, in Fig. 4k for *s* $=$ 2.1 nm, and in Fig. 4l for *s* $=$ 1.7 nm. The plots from the denoised images agree well with those from the reference-measurement images. The results explicitly indicate the usefulness of noise reduction by WHMM.

**Fig. 5:**
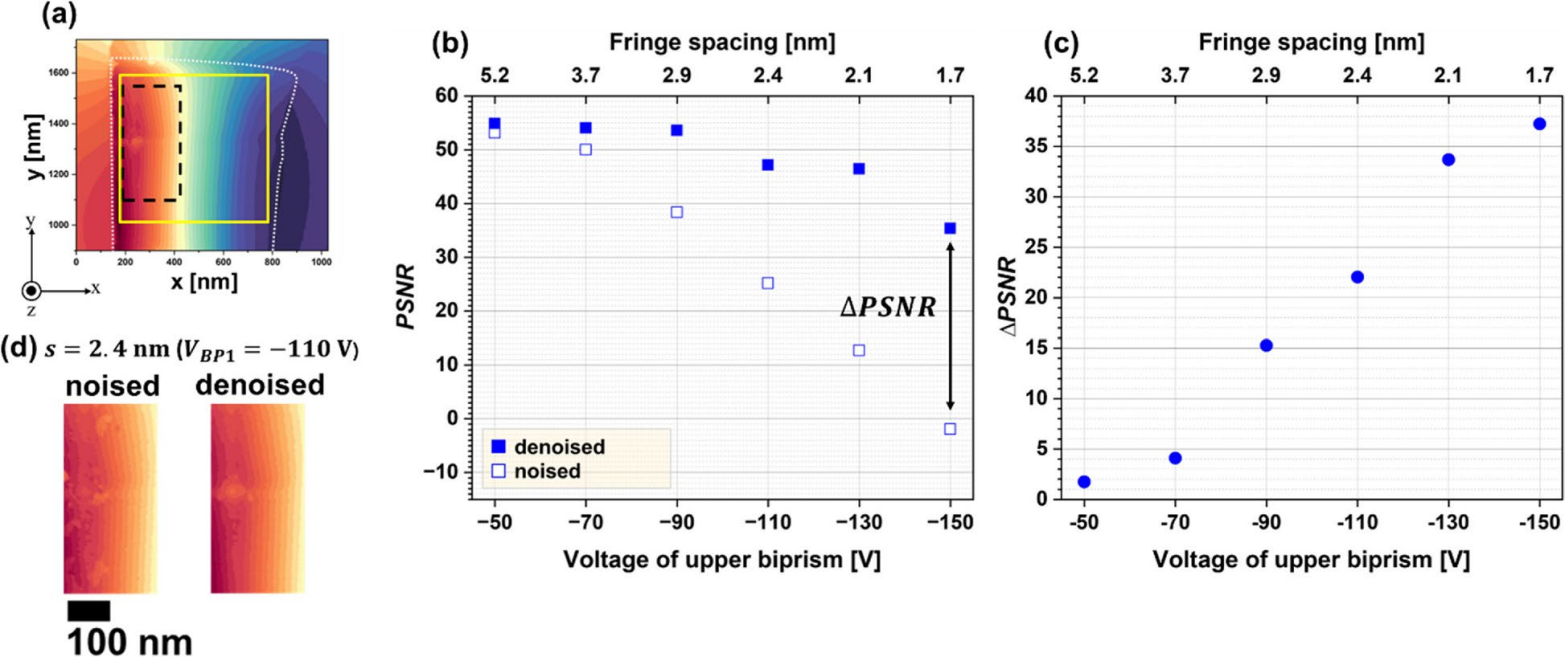
Evaluation of the denoising effect using peak signal-to-noise ratio (*PSNR)*. **a** Reference-measurement phase image for the fringe spacing *s* $=$ 2.4 nm. *PSNR* was determined for the area indicated by the yellow lines. **b** *PSNR* as functions of the upper biprism voltage *V*_*BP1*_ (lower horizontal axis) and the fringe spacing (upper horizontal axis). Open and closed squares indicate *PSNR* values determined from the original phase images (including noise) and the noise-reduced (denoised) phase images, respectively. **c** Difference between the *PSNR* for the denoised images (closed squares) and original phase images (open squares) as function of *V*_*BP1*_ (lower horizontal axis) and *s* (upper horizontal axis). **d** Noised (left) and denoised (right) images obtained at *s* $=$ 2.4 nm. The black dotted lines in (**a**) indicate the enlarged areas in (**d**)

For further examination of the noise reduction, the peak signal-to-noise ratio (*PSNR*) defined by Eq. (3) was calculated for the denoised images (Arora et al. 2008):

3 $$PSNR=20\cdot {{\text{log}}}_{10}\left(255/\sqrt{1/MN{\sum }_{i=1}^{M}{\sum }_{j=1}^{N}{\left(I\left(i,j\right)-G\left(i,j\right)\right)}^{2}}\right)$$

where $I\left(i,j\right)$ represents the intensity of either the original (without denoising) or denoised images measured at pixel positions $\left(i,j\right)$. *M* and *N* represent the pixel size; in this study, *M* = 536 for $i$ (in *x* direction) and *N* = 536 for $j$ (in *y* direction). $G\left(i,j\right)$ represents the intensity of the reference-measurement image measured at pixel position $\left(i,j\right)$. The *PSNR* was calculated for the area enclosed by the yellow lines in Fig. 5a, which is identical to Fig. 4a. Figure 5b shows the *PSNR* as a function of the voltage applied to the upper biprism *V*_*BP1*_ (*i.e.*, lower horizontal axis) and fringe spacing *s* (*i.e.*, upper horizontal axis). The open squares represent the *PSNR* determined for the original phase images without noise reduction, whereas the closed squares represent values for the denoised images. Regarding the open squares representing noisy images (without WHMM denoising), the *PSNR* continued to decrease with decreasing fringe spacing, because the fringe contrast decreased with decreasing fringe spacing. Note that the plot at *s* $=$ 1.7 nm (for open square) takes a negative value at *PSNR* $=-$1.9. This result indicates that the signal is much weaker than the noise under these conditions. When noise reduction by WHMM was applied, the *PSNR* increased under all fringe spacing conditions, as shown in the closed square in Fig. 5b. Importantly, the improvement in *PSNR* [*i.e.*, $\Delta$*PSNR*, defined by the difference between the *PSNR* for the denoised images (closed square) and noised images (open square)] was significant for the observations with small fringe spacing. As demonstrated in Fig. 5c, $\Delta$*PSNR* continues to increase as the fringe spacing is reduced. It is interesting that the *PSNR* at *s* $=$ 1.7 nm (for denoised image) becomes comparable to the value at *s* $=$ 2.9 nm (for noisy image) as a result of noise reduction by WHMM. Although the visibility of the fringes (responsible for the sensitivity of phase detection) deteriorates when the fringe spacing (responsible for the spatial resolution) is reduced, noise reduction helps improve the fringe contrast. Thus, noise reduction using the WHMM is advantageous for electron holography with a high spatial resolution. To see the effectiveness of noise reduction on the magnetic domain analysis, the left panel of Fig. 5d shows the original reconstructed phase image (labelled by “noised”) which was obtained at the condition of *s* $=$ 2.4 nm. The field of view is identical to the area closed by the dotted in Fig. 5a. Although this area represents a single magnetic domain that was magnetized in the* -y* direction, the original image shows several phase discontinuities (*i.e.*, patches in the phase images) due to the imperfect phase retrieval using a noised electron hologram. However, when the noise reduction was applied, the phase discontinuities were removed as shown in the right panel of Fig. 5d (labelled by “denoised”). The residual patch in the right panel (denoised image), which is present at the center/left position in the field of view, represents the area the thickness of which was reduced in the process of sample preparation. Thus, the noise reduction helps us examine the magnetic domain structure and/or magnetic flux density using electron holography.

There are several methods of transmission electron microscopy that allow magnetic domain observations: *i.e.*, Lorentz microscopy, differential phase contrast (DPC) with scanning transmission electron microscopy (STEM), electron holography, etc. Regarding the advantage of electron holography, this method is useful for the magnetic flux density measurement from a grain boundary (GB) region (Murakami et al. 2014; Cho et al. 2020), while tailoring of the grain boundary region is essential for the coercivity improvement. Thus, the noise reduction using WHMM, which is applied to electron holography, can be a powerful tool in the research and development of Nd-Fe-B permanent magnets.

## Conclusion

We demonstrated the effectiveness of noise reduction when the WHMM is applied to phase retrieval by electron holography. Electron holograms were acquired from thin-foiled Nd_2_Fe_14_B crystals with various fringe spacings. The narrow fringe spacing, which can provide a high spatial resolution of the phase analysis, reduces the visibility of the holograms, resulting in artificial jumps of the phase shift in the reconstructed phase image. When WHMM denoising was applied to both real and imaginary parts of the complex images obtained in the process of phase retrieval, the undesired patches (due to the artificial phase jump) could be suppressed in all the observations acquired in the range of fringe spacing from 1.7 nm to 5.2 nm. Particularly, the denoising effect is significant for observations with narrow fringe spacing. The visibility of the interference fringes in holograms deteriorates when the fringe spacing is reduced, whereas a narrow spacing is required for observations at a high spatial resolution. Denoising holograms are a promising approach to solve this technical problem.

## Data Availability

Not applicable.

## Abbreviations

TEM: Transmission electron microscopy
WHMM: Wavelet hidden Markov model
FFT: Fast Fourier transform
FIB: Focused ion beam
SEM: Scanning electron microscope
FFT^-1^: Inverse Fourier transform
MTF: Modulation transfer function
PSNR: Peak signal-to-noise ratio
DPC: Differential phase contrast
STEM: Scanning transmission electron microscopy
